# Proteomic approaches for the study of tissue specific effects of 3,5,3′-triiodo-L-thyronine and 3,5-diiodo-L-thyronine in conditions of altered energy metabolism

**DOI:** 10.3389/fphys.2014.00491

**Published:** 2014-12-17

**Authors:** Elena Silvestri, Maria Coppola, Federica Cioffi, Fernando Goglia

**Affiliations:** Dipartimento di Scienze e Tecnologie, Università degli Studi del SannioBenevento, Italy

**Keywords:** iodothyronine, metabolism, proteomics, obesity, mitochondrion

## Abstract

In vertebrates and, specifically, in mammals, energy homeostasis is achieved by the integration of metabolic and neuroendocrine signals linked to one another in an intricate network hierarchically responding to the tight modulating action of hormones among which thyroid hormones (THs) play a central role. At the cellular level, 3,5,3′-triiodo-L-thyronine (T3) acts mainly by binding to specific nuclear receptors (TRs) but actually it is becoming more and more evident that some T3- actions are independent of TRs and that other iodothyronines, such as 3,5-diiodo-L-thyronine (T2), affect energy metabolism and adiposity. In the postgenomic era, clinical and basic biological researches are increasingly benefiting from the recently developed new omics approaches including, among the others, proteomics. Considering the recognized value of proteins as excellent targets in physiology, the functional and simultaneous analysis of the expression level and the cellular localization of multiple proteins can actually be considered fundamental in the understanding of complex mechanisms such as those involved in thyroid control of metabolism. Here, we will discuss new leads (i.e., target proteins and metabolic pathways) emerging in applying proteomics to the actions of T3 and T2 in conditions of altered energy metabolism in animal tissues having a central role in the control of energy balance.

## Introduction

Strong evidence supports an essential role for thyroid hormones [THs, 3,5,3′,5′-tetraiodo-L-thyronine (T4), and 3,5,3′-triiodo-L-thyronine (T3)] in the physiological regulation of whole-body energy balance and metabolism in homoeothermic species and, specifically, in mammals. Moreover, THs control a bulk of physiological processes such as growth, development, and metabolic rate (for a recent review on thyroid hormone signaling in energy metabolism, see Brent, 2012; Davis et al., 2013; López et al., 2013; McAninch and Bianco, 2014; Mullur et al., 2014). However, the network of events involved in the actions of THs is complicated and still incompletely understood. Integrative omic approaches, such as transcriptomics and proteomics, hold great promise and actually are having increasing applications in the study of complex biological systems. As far as it concerns the metabolic actions of THs, a large amount of data is available from transcriptomic studies which have been quite extensively performed to identify T3-target genes in several models (for review see, Silvestri et al., 2011a, and references therein) actually providing a cornucopia of novel information on the regulation of transcription by THs (e.g., the potential of T3 to regulate miRs, Visser et al., 2009). However, the intrinsic nature of these studies provided no information concerning the status of the corresponding encoded proteins, which, indeed, enable to assess the programs actually executed. On the other hand, proteomic approaches [such as, among the others, two-dimensional gel electrophoresis (2-DE) and mass spectrometry (MS)], allow the simultaneous measurement and comparison of the expression levels of hundreds of proteins as well as the identification of other aspects of protein functions (i.e., post-translation modifications), giving rise to a fuller understanding of cellular functions (for recent review, see Johnson and White, 2012; Feeney and Schöneich, 2013; Zhang et al., 2013). However, intrinsic limitations inherent to the more classical approaches—including, among the others, difficulties in the resolution of proteins with extreme isoelectric point values, loss of very hydrophobic proteins, absence of proteins of high and low molecular weights and poor resolution of low-abundance proteins—cannot be ignored (Sriharshan et al., 2014; and for review see, Wittig and Schägger, 2009; Silvestri et al., 2011a,b). In this review, we will outline the new leads emerging from the application of proteomic analyses to the actions of thyroid hormones in the regulation of energy balance in conditions of altered energy metabolism.

## Proteomic analysis pertaining to the actions of T3

Hypothyroidism and hyperthyroidism are two pathological conditions in which energy metabolism is altered leading to hypo- and hyper-metabolic states, respectively. Metabolically active tissues, such as liver and skeletal muscle, retain the ability to respond, to counteract and to adapt to such alterations by modifying gene/protein expression patterns.

The first application of proteomic tools to the modulations that T3 exerts *in vivo* over tissue proteins can be traced back to 1981. Analyzing the liver of normal, thyroidectomized, and thyroidectomized plus T3 treated rats, by using classical 2-DE, it has been shown that changes in the thyroid state significantly affect the composition of the hepatic nucleoproteins (Nikodem et al., 1981).

To obtain a more comprehensive identification and characterization of molecular events/pathways associated with altered thyroid states, more recently, a high-resolution differential proteomic analysis, combining 2-DE and subsequent matrix-assisted laser desorption/ionization time-of flight mass spectrometry (MALDI-ToF MS), was performed, thus providing the first systematic identification of T3-induced changes in the protein expression profiles of liver and skeletal muscle of hypothyroid rats (Silvestri et al., 2006, 2007).

In the liver, among the 600 detected spots, 53 proteins (8% of the analyzed proteome) resulted to be significantly affected by T3 treatment. On the base of their identity, the unambiguously identified proteins were classified as involved in substrate (e.g., aldehyde-dehydrogenase and α-enolase) and lipid (e.g., short chain-specific acyl-CoA dehydrogenase and 3-ketoacyl-CoA thiolase) metabolism, energy metabolism (e.g., ATP synthase α-chain), detoxification of cytotoxic products (e.g., catalase and glutathione-S-transferase), calcium homeostasis (senescence marker protein 30), amino acid catabolism (arginase-1), and the urea cycle (ornithine carbamoyltransferase). Interestingly, 76% of these proteins were down-regulated in hyperthyroid rats vs. hypothyroid ones. These proteomic data gave new support to the transcriptome analyses revealing the idea that negative regulation by T3 on gene expression might be much more prevalent than previously thought (Feng et al., 2000). Among the down-regulated proteins, there were ornithine carbamoyltransferase, arginase 1, the peroxisomal catalase, and the cytoplasmic glutathione-S-transferase. This allowed to deeper understand how the thyroid state, in the liver, modulates urea and ammonia production (Marti et al., 1988) and promotes oxidative stress (for recent review, see Videla, 2010).

On the other hand, among the few up-regulated proteins, α-enolase resulted to be strongly induced (+240%) by T3 administration. This further supported the notion that T3 stimulates gluconeogenesis and glucose production in the liver thus opposing to the action of insulin (for recent review, see Brenta, 2011).

Concerning skeletal muscle, the whole-cell protein content of gastrocnemius muscle was analyzed. With the detection limits set, a proteome of about 200 spots was obtained. 33 protein spots (15% of the analyzed proteome) resulted to be significantly affected by T3 treatment. When comparing hyperthyroid vs. hypothyroid rats, 70% of the unambiguously identified proteins (20 protein spots) were up-regulated. The largest group of affected proteins was involved in substrate (e.g., pyruvate kinase muscle isozyme and malate dehydrogenase) and energy metabolism (e.g., creatine kinase M-type and ATP synthase beta chain), another important group was represented by stress-induced proteins (HSPs), and the remainder were implicated in structural features or gene expression (transcription, translation) (e.g., chromodomain-helicase-DNAbinding protein 1 and eukaryotic translation initiation factor 3 subunit 10). Importantly, the thyroid state resulted to induce simultaneous changes in the expression levels of proteins involved in both structural and metabolic features of the gastrocnemius muscle. Specifically, hypothyroidism and hyperthyroidism were found to induce a structural and metabolic shift toward a slower and a faster phenotype, respectively (Silvestri et al., 2007). Indeed, in accordance with a predominant expression of myosin heavy chain (MHC) Ib over MHC IIb in hypothyroidism and a reversal of this ratio after T3 administration, the expression level of myosin regulatory light chain 2, typical of slow-twitch fibers, was strongly increased in hypothyroidism, with hyperthyroidism significantly reducing it. Coherently, and in accordance with a generally increased metabolic dependence on glycolysis in hyperthyroidism, β-enolase, pyruvate kinase, and triosephosphate isomerase protein levels were significantly increased following T3 treatment.

All together, the so far described data allowed to obtain an at glance evaluation of the effects elicited by T3 in hypothyroid rats, highlighting the tissue-specific proteomic response: in the liver T3 produces a general down regulation of affected proteins likely modulating substrate metabolism (i.e., aminoacid catabolism); in the gastrocnemius muscle T3 produces a more pronounced effect of up-regulation likely promoting energy metabolism and glucose utilization.

An even more recent study was published in which proteomic approaches were used to obtain new information on the *in vivo* actions of T3. Specifically, the early changes induced in rat liver by a single mitogen dose of T3 were characterized (Severino et al., 2011). Many enzymes, directly or indirectly involved in energy metabolism and oxidative stress, were identified among the differentially expressed proteins furnishing new insight into the mechanisms by which T3 induces hepatocyte proliferation.

These studies, as a whole, allowed the identification of proteins previously not known to be regulated by T3 and might prompt the scientific community to go further with proteomic-based approaches to increase the awareness of the multiple cell processes and signaling pathways involved in the effects of such iodothyronine.

## Proteomic analysis pertaining to the actions of T2

Overweight and adiposity lead to impaired energy balance with several other metabolic disturbances taking place. T3 exerts both hypolipidemic and hyperlipidemic effects due to its control of lipolytic and lipogenic pathways. Interestingly, accumulating evidence has indicated that several metabolic effects of THs can be attributed to endogenous metabolites of T3 that actually can be considered new discovered arms by which the thyroid gland can control whole body energy homeostasis (Goglia, 2005; Moreno et al., 2008; Piehl et al., 2011; Senese et al., 2014). 3,5-diiodo-l-thyronine (T2) is receiving particular attention in view of its specific excito-metabolic actions (for recent review, see Coppola et al., 2014; Senese et al., 2014 and references within).

The effects and mechanisms underlining the actions of T2 have so far been studied both *in vivo* and *in vitro* (Scapin et al., 2010; Grasselli et al., 2011, 2012) models (for recent review, see Silvestri et al., 2013; Vergani, 2014). Up to now, a controversy exists as far as it concerns the nature of such mechanisms and the possibility that T2 might exert some of its effects in a TR-dependent manner (Mendoza et al., 2013; Jonas et al., 2014; Navarrete-Ramírez et al., 2014; Orozco et al., 2014). However, it cannot be excluded that the putative interaction of T2 with nuclear TRs, at least in mammalian species, might be a consequence of the high doses used (Goldberg et al., 2012; Jonas et al., 2014).

Of the currently described *in vivo* effects of T2, a particular physiological and pharmacological relevance appears to be associated with those that we can define as hypolipidemic and anti-steatotic effects, that have been described in several animal models of overweight and thus of altered energy metabolism [high fat (HFD) or high cholesterol diet-fed rodents] (Lanni et al., 2005; Grasselli et al., 2008; Mollica et al., 2009; de Lange et al., 2011; Moreno et al., 2011; Goldberg et al., 2012; Jonas et al., 2014) and in humans too (Antonelli et al., 2011). Importantly, a more recent study has reported that T2 administration stimulates energy expenditure and reduces body mass gain also in standard diet fed aging rats (Padron et al., 2014).

As far as it concerns the anti-steatotic effect of T2 elicited in the liver of HFD rats (at the pharmacological but not thyrotoxic dose of 25 μg/100 g body weight), to further identify candidate molecules as well as molecular/biochemical pathways linking fat consumption, the pathogenesis of hepatic steatosis, and mitochondrial functions, a high-resolution differential proteomic analysis [by combining 2-DE, MALDI-ToF MS, Blue Native-PAGE (BN-PAGE) and *in silico* analysis] was performed (Silvestri et al., 2010).

Data analysis demonstrated that the steatotic effect of HFD, and the anti-steatotic effect of T2-treatment are strictly associated with altered expression levels of several proteins and enzymes involved in key liver metabolic pathways. These pathways included: fatty acid metabolism [e.g., carnitine O-palmitoyltransferase (CPT) 2 and long-chain fatty acid-CoA ligase], ketone-bodies [e.g., hydroxymethylglutaryl-CoA (HMG-CoA) synthase] and energy metabolism (e.g., ATP synthase subunit alpha), amino acid and nitrogen metabolism (e.g., glutamate dehydrogenase 1), the urea cycle (e.g., carbamoyl-phosphate synthase), the stress response (e.g., HSP60 and catalase) and protein turnover (e.g., proteasome component C2 and proteasome iota chain). Importantly, after T2-treatment, the majority of these proteins exhibited alterations in their expression level that were opposite to those observed in HFD and normalized vs. those observed in standard diet fed control rats (Silvestri et al., 2010). For example, long-chain fatty acid-CoA ligase and ATP synthase subunit alpha, both having a central role in lipid biosynthesis and energy metabolism, were significantly up-regulated by HFD (thus positively correlating with the steatotic condition present in HFD rats) and normalized in their expression levels by T2 treatment, thus highlighting some of the biochemical bases of the hypolipidemic effect of T2. The obtained proteomic data were corroborated by the *in silico* analysis (i.e., pathway and network analysis by using the Ingenuity® Systems, www.ingenuity.com). Of note, the network concerning the effects of T2 contained two external coupling namely HMG-CoA synthase and CPT, supporting, once again, the centrality of lipid metabolism in the action of T2 in the liver. Moreover, as mitochondria appeared to be a major target for the metabolic and energy adaptations induced by fat-overload, and displayed a significant response, in terms of their proteome, to T2-treatment, a BN-PAGE-based approach was also used to characterize the profile of the liver OXPHOS and to measure their individual in-gel activities. In summary, T2 induced vs. HFD, an enhancement of OXPHOS activities (complex I, II, and IV) well suggesting reduced oxidative damage to the respiratory chain itself and a success in efficiently catabolizing the extra load of fatty acids, likely “sparing” other metabolically active tissue, specifically the skeletal muscle, counteracting, at the same time, the fat-induced insulin resistance (IR) attributable to HFD (de Lange et al., 2011). To test this hypothesis, by using the same animal model as in Silvestri et al. (2010), an integrated approach was designed to assess the effects of T2 on skeletal muscle insulin sensitivity and protein profile (Moreno et al., 2011). Schematically, without inducing sarcopenia, T2 administration to HFD rats produced in gastrocnemius muscle an increase in: type II (glycolitic) fibers, glucose transporter 4 (GLUT4) membrane content, glucose utilization (through Akt activation), and glycolytic enzymes activity, while preventing the HFD-induced increase in fatty acid uptake (through FAT/CD36) and intramyocellular lipid (triglyceride) accumulation. The 2-DE/nano-liquid chromatography-electrospray ionization-linear ion trap-tandem mass spectrometry (nLC-ESI-LIT-MS/MS)-based analysis revealed that T2 treatment, while inducing a shift toward a fast phenotype, significantly altered the gastrocnemius muscle protein expression profile of HDF rats. In particular, structural proteins such as the fast isotypes of myosin light chains (i.e., MLC1f, MLC2f) and the tropomyosin α chain fast increased significantly following T2 treatment whereas the content of the slow isotypes MLC1s, MLC2s, and the tropomyosin α chain slow decreased. Coherently, in T2-treated HFD rats, other glycolytic enzymes (i.e., α- and β-enolase, and triosophosphate isomerase) were up-regulated while enzymes involved in oxidative metabolism (i.e., carbonic anhydrase III and myoglobin) were significantly down-regulated vs. HFD control rats (Moreno et al., 2011).

Overall, these data indicated, for the first time, that T2, at least at the used dose and in rats, without thyrotoxic effects, can significantly impact the proteome profile of responsive tissues, such as liver and skeletal muscle, thus ultimately influencing fuel utilization, and energy metabolism in a functional cross-talk between target organs. In particular, in terms of tissue specific effects of T2, the liver might be mainly involved in lipid metabolism derangement while the skeletal muscle might be mainly implicated in glucose utilization.

## Conclusions and perspectives

**Figure 1:**
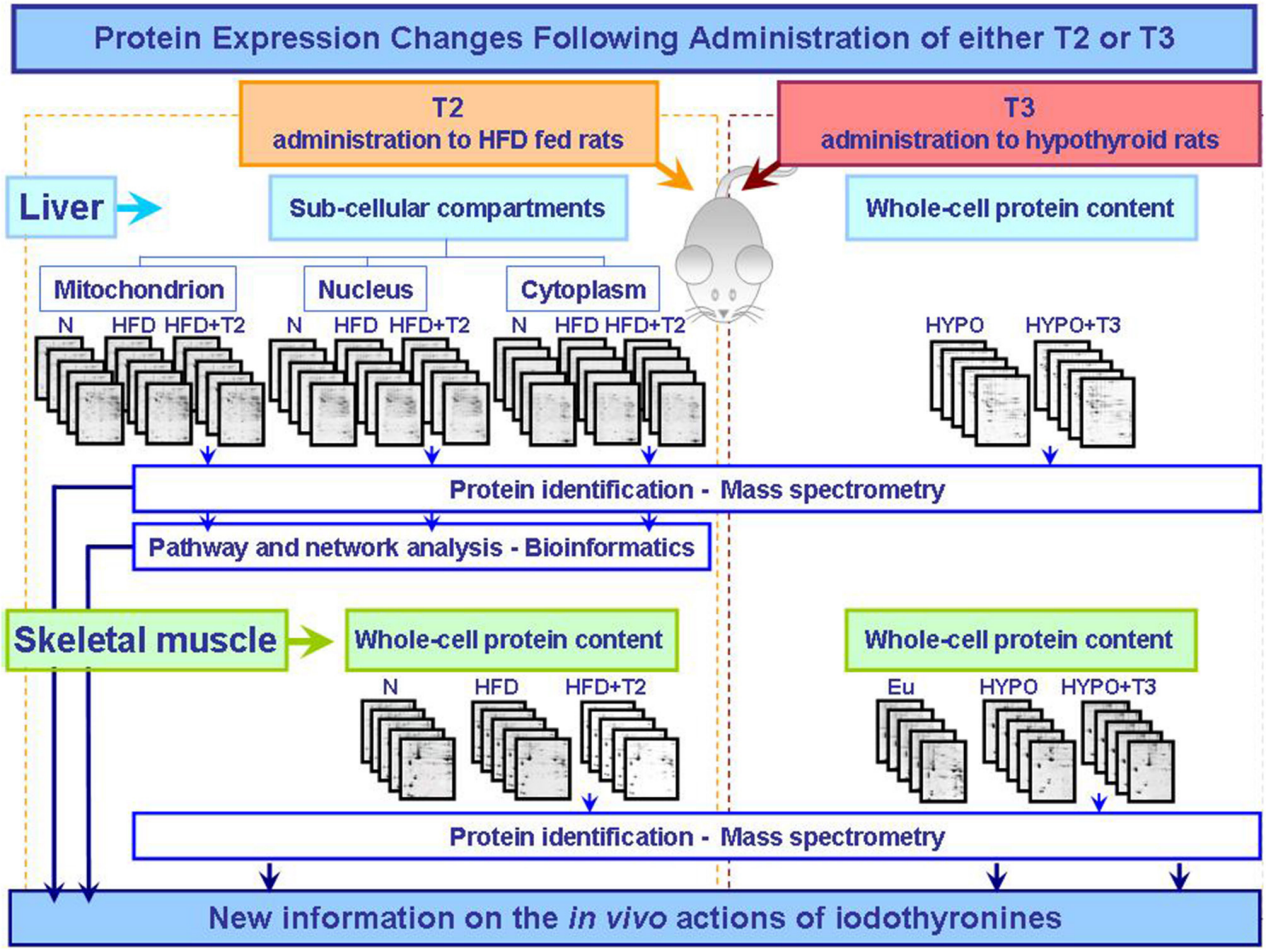
**Synoptic of the workflow of the proteomic approaches so far utilized to obtain new information on the actions that T2 and T3 exert *in vivo* in key metabolically active tissues central in the control of energy balance**. The described studies were performed as integrated approaches including 2D-E, mass spectrometry, and bioinformatic tools (Silvestri et al., 2006, 2007, 2010; Moreno et al., 2011). Abbreviations: N, standard diet fed control rats; HFD, high fat diet fed rats; HFD+T2, high fat diet fed rats treated with T2; Eu, euthyroid rats; Hypo, hypothyroid rats; Hypo+T3, hyperthyroid rats.

The biochemical and cellular mechanisms that underlie tissue specific actions of T3 and T2 are only beginning to be elucidated. However, the proteomic studies so far conducted separately analyzed the effects of T3 and T2 in different states of altered energy balance: changed thyroid state and over-nutrition, respectively (Figure 1). To further characterize and compare the molecular and biochemical pathways that underlie T3 and T2 metabolic actions, T3 and T2 themselves should be used in the same experimental design in comparative approaches so to highlight putative common effects or iodothyronine-specific one.

### Conflict of interest statement

The authors declare that the research was conducted in the absence of any commercial or financial relationships that could be construed as a potential conflict of interest.
